# Telephone-Delivered Interventions for Suicide Prevention in Schizophrenia and Related Disorders: A Systematic Review

**DOI:** 10.1192/j.eurpsy.2023.1184

**Published:** 2023-07-19

**Authors:** L. Comendador Vázquez, A. I. Cebrià, A. Sanz, V. Pérez, D. J. Palao

**Affiliations:** 1 Mental Health Service. Hospital Universitari Parc Taulí, Corporació Sanitaria Parc Taulí, Sabadell; 2 Department of Psychiatry and Forensic Medicine, Faculty of Medicine, Universitat Autònoma de Barcelona, Bellaterra; 3 Centro de Investigación Biomédica en Red de Salud Mental (CIBERSAM), Madrid; 4 Department of Clinical and Health Psychology, Faculty of Psychology; 5 Department of Basic, Developmental and Educational Psychology; 6 Stress and Health Research Group (GIES), Universitat Autònoma de Barcelona, Bellaterra; 7 Institut de Neuropsiquiatria i Addiccions (INAD), Institut Hospital del Mar d’Investigacions Mèdiques (IMIM), Parc de Salut Mar, Barcelona, Spain

## Abstract

**Introduction:**

Suicide is a health concern among individuals diagnosed with schizophrenia. Telehealth technology has become an emerging intervention that may afford opportunities for reaching this at-risk group. Consideration of the implementation of telehealth systems in the treatment of patients diagnosed with schizophrenia and suicidal behavior calls for a review of the evidence.

**Objectives:**

The present aim was to explore the literature on the effectiveness of suicide prevention telephone delivered interventions among patients with schizophrenia and related disorders.

**Methods:**

The bibliographic search was performed in the electronic databases PubMed, PsycInfo, Scopus, and Web of Science following PRISMA guidelines. Two reviewers independently conduct screenings, data extraction and methodological quality assessment. A total of 352 articles were retrieved, of which five studies met the eligibility criteria.

**Results:**

Based on the limited data available, the use of modalities involving telephone contacts appears to be feasible in patients with schizophrenia and suicidal behaviors. In addition, preliminary evidence suggests this system appears to reduce suicidal ideation.

**Conclusions:**

The current data presented here reflect an early stage of effectiveness of telephone-delivered interventions targeted at suicide prevention in patients with schizophrenia. Further research is needed to design evidence-based future interventions and to determine whether this approach can improve patient outcomes.

Fundings: This work was supported by the Instituto de Salud Carlos III (grant number: PI19/01484 SURVIVE).

Acknowledgements: SURVIVE project (PI19/01484).

Keywords: schizophrenia, suicide, telehealth, monitoring.

**Disclosure of Interest:**

None Declared

